# Latent Diphtheria

**Published:** 1907-08

**Authors:** 


### LATENT DIPHTHERIA.

Myer Solis-Cohen concludes that: The prevalence of diphtheria is due to the lack of control over latent cases of diptheria and over the so called "carrier" cases. Diptheria may occur in a latent forms without pseudomembrane and with only slight symptoms. Latent cases of diphtheria should be isolated until two successive negative cultures have been obtained. All cases of sore throat should be reported to the health authorities and should be examined bacteriologically. Infected "contacts" should be excluded from school or work, and should not be permitted to frequent public places until two sucessive cultures have proven negative. All who have been in contact with a diphtheria patient, whether at home, at school, or at work, should be examined bacteriologically. Disinfection of fomites and terminal disinfection of rooms and their contents is insufficient and reliance there treacherous. Animate carriers of infection are more dangerous than the inanimate.—Jour. A. M. A., July 6th, 1907.

